# The Readiness of Psychiatrists to Implement Psychedelic-Assisted Psychotherapy

**DOI:** 10.3389/fpsyt.2021.743599

**Published:** 2021-11-26

**Authors:** Lisa A. Page, Ahmad Rehman, Habib Syed, Kathryn Forcer, Graham Campbell

**Affiliations:** ^1^Brighton and Sussex Medical School, Brighton, United Kingdom; ^2^Sussex Partnership National Health Service (NHS) Foundation Trust, Worthing, United Kingdom; ^3^Brighton and Sussex University Hospitals National Health Service (NHS) Trust, Brighton, United Kingdom; ^4^Small Pharma Ltd., London, United Kingdom

**Keywords:** psychedelic-assisted psychotherapy, psychiatrists, attitudes, implementation, knowledge, mixed-methods, cross-sectional survey

## Abstract

**Introduction:** Psychedelic-assisted psychotherapy is a promising approach in psychiatry; evidence is growing and it may not be long before mainstream services are expected to offer it to selected patients. This pilot study examined the attitudes and knowledge of NHS psychiatrists of all levels towards psychedelic-assisted psychotherapy and explored potential barriers and facilitators to its implementation.

**Methods:** A mixed-methods approach was adopted, using a cross-sectional survey and focus groups. All psychiatrists in one NHS mental health trust were approached by email to participate. The survey was analysed using a simple descriptive approach and thematic analysis was used for the focus groups.

**Results:** Eighty-three (25.7%) psychiatrists participated in the survey. All psychiatrists were familiar with one or more psychedelic substances. Although 77.2% felt that there should be a role for controlled or therapeutic use of psychedelics, trainees appeared better informed than non-training grade psychiatrists. Psychiatrists of all grades did not feel prepared to participate in the delivery of psychedelic-assisted psychotherapy. Thematic analysis of the focus groups identified three main themes in relation to psychedelic-assisted psychotherapy: “*need for knowledge*,” “*openness to change*,” and “*uncertainty*.”

**Discussion:** NHS psychiatrists are positive about the potential for psychedelic-assisted therapy to advance psychiatric practise. However, psychiatrists are lacking in confidence or preparedness to implement this treatment should it become a mainstream option and significant training needs were identified. Thematic analysis highlighted the need for societal shifts as well as professional ones.

## Introduction

Psychedelic-Assisted Psychotherapy (PAP) offers a potentially promising and novel therapeutic approach in psychiatry (1–3). The evidence base for the use of PAP in mental disorders is growing (4–6) against a backdrop of considerable public interest https://www.bbc.co.uk/mediacentre/proginfo/2021/19/the-psychedelic-drug-trial. There is particular interest in the use of PAP in depressive disorders (7, 8), cancer-related anxiety (9) and alcohol dependence (10). Trials are ongoing in these and other mental disorders and psilocybin was designated a “breakthrough therapy” by the US Food and Drug Administration (FDA) in 2019.

Psychedelics are potent serotonergic hallucinogens that induce perceptual changes and elicit altered states of consciousness; they are understood to act *via* the full or partial agonism of cortical 5-hydroxy tryptamine 2A receptors (3, 11–13). Increasingly psychedelics are seen as safe medicines, with limited abuse potential, and a rapid yet novel action (3).

### What Is Psychedelic-Assisted Psychotherapy?

PAP typically entails a series of psychotherapeutic sessions with three distinct phases: induction, psychedelic and integrative phases (14, 15). The induction sessions focus on the therapeutic relationship and the patient is prepared for the psychedelic session to come. The psychedelic session, of which there is usually one but sometimes more, involves the patient ingesting a psychedelic substance in the presence of therapist guides. The therapist guides are typically mental health professionals—psychologists, psychotherapists or psychiatrists—who monitor and if necessary, guide the patient through the experience. The number of psychedelic sessions and the psychedelic dose varies depending on the research design and the type of psychedelic used. During the subsequent integrative sessions, the therapists help facilitate patient understanding and interpretation of the psychedelic experience with aim of achieving long-term change (14). To date, psychiatrists have played several important roles in the (research) deployment of PAP, including: research leadership, patient selection, risk management, prescriber and therapist guide.

### Why Might PAP Be Offered Within Mainstream Services?

PAP offers an alternative and novel approach compared to existing pharmacological or psychological approaches (3). As the evidence builds for the effectiveness of PAP in differing mental disorders, health services may soon be routinely expected to offer it—especially for patients who have not responded to existing treatments and remain functionally impaired. Despite this possibility, it is not known whether the workforce who would provide PAP, including psychiatrists, is prepared to do so.

### Are Psychiatrists Ready for PAP?

To our knowledge, there has been only one previous study looking at the psychiatrists' attitudes towards the use of psychedelics (16). In 2018, Barnett et al. surveyed members of the American Psychiatric Association about their views on the use of hallucinogens; they found that 80.5% of respondents felt there should be more research in relation to hallucinogens and psychiatric disorder, but less than a third felt that hallucinogens were likely to improve outcomes when used with psychotherapy. A substantial minority (24.5%) felt that hallucinogens were unsafe even under medical supervision. This study also showed that trainee psychiatrists and male psychiatrists tended to be less concerned about the risk of hallucinogens and more optimistic about their potential.

Since 2018, the evidence for PAP has strengthened and we were keen to build on the work of Barnett et al. to find out whether British psychiatrists held similar attitudes to US psychiatrists. Most British psychiatrists work in a nationalised healthcare system (the NHS), unlike their US counterparts. Furthermore, we sought to understand what psychiatrists saw as the potential difficulties of implementing PAP, which represents a considerable paradigm shift compared to current practise.

The primary objective of this pilotstudy was to investigate the awareness, knowledge and attitudes of NHS Psychiatrists towards the possible introduction of PAP within a nationalised healthcare system. Secondary objectives included identifying potential barriers and facilitators to the adoption of PAP within psychiatric practise.

## Methods

A mixed methods approach was adopted by using both a (quantitative) cross-sectional survey and (qualitative) focus groups. We restricted our study to questions about classic hallucinogens, such as LSD, psilocybin, mescaline, and deliberately did not include questions about related drugs such as MDMA or ketamine.

### Cross-Sectional Survey

First, an invitation to participate in an e-survey was sent by email to all Psychiatrists within one NHS mental health trust in England in January 2021. The mental health trust provides mental health and learning disability services across all ages to a population of nearly 1.7 million people from a large and geographically diverse area. A reminder email was sent ~2 weeks after the first invite to maximise response. The survey was developed, refined and piloted by the research team with the aim of eliciting psychiatrists' views on key aspects of PAP. Specifically, the survey examined awareness, knowledge and attitudes towards PAP and elicited psychiatrists' views as to how confident they would feel to refer patients for PAP or provide PAP within their practise. In addition, the survey elicited psychiatrists' opinions on current effectiveness of psychotherapeutic and pharmacological approaches in the treatment of depression, anxiety and substance use disorders.

In order to include a representative sample of psychiatrists from different sub-specialties and with varying levels of experience and seniority, all psychiatrists from one NHS mental health trust were invited to participate. Therefore, psychiatrists in training, as well as those working permanently in specialty doctor and consultant posts were included in the sampling frame. The survey was hosted on Qualtrics and all responses were anonymous.

Basic descriptive statistics were used to analyse the survey data. An a-priori decision was taken to investigate whether age was a contributing factor in psychiatrists' decision to be more or less prepared to get involved with PAP, we therefore divided psychiatrists into three age groups (i.e., ≤ 34, 35–54, and ≥55 years) and used the chi-squared test to compare their responses.

### Focus Groups

Second, we convened two focus groups for psychiatrists from the same NHS trust. The invitation to participate in the focus groups was embedded in the survey invite and again at the end of the survey—the sample was therefore purposive. The focus groups were facilitated by two members of the research team (KF and HS) and were hosted on a virtual platform due to Covid-19 restrictions. No previous knowledge of PAP was required and the facilitators spent a short period at the beginning of each focus group describing PAP and how PAP was being used in research settings. The research team developed a schedule for use with the focus groups, which ensured that psychiatrists' attitudes about PAP were explored in depth as were potential barriers and facilitators to the adoption of PAP in psychiatric practise.

The focus groups were audio-recorded and then transcribed before the audio files were destroyed. All contributions were anonymised and no personally identifiable information was stored. The transcripts were analysed using Thematic Analysis by two members of the research team (KF & LP). The analytic approach was based on the work of Braun and Clarke (17).

### Ethical Approval and Sponsorship

NHS ethical approval was not required for the study, but good ethical procedures were followed throughout. The study was sponsored by Sussex Partnership NHS Foundation Trust.

## Results

### Cross-Sectional Survey

The questionnaire was sent out to a total of 323 psychiatrists, of whom 48 were trainees (i.e., they were rotating through core training or higher specialist trainee posts in psychiatry) and 275 were consultants or specialty doctors (i.e., they were working in permanent, non-training grade posts). In total, 83 (25.7%) responses were received, of which 63 (75.9%) were either consultants or specialty doctors and 20 (24.1%) were trainees. A greater proportion of trainees responded to the survey than non-training grade doctors (41.7 vs. 22.9%). Just over half of survey participants were aged between 35 and 54 years (*n* = 48; 57.8%), whilst 17 (20.5%) were aged over 54 years and 14 (16.9%) were aged 34 or younger.

Most sub-specialties were represented. Participants' most common specialty area was Community General Adult Psychiatry (43.4%) followed by Old Age Psychiatry (21.7%) and Inpatient General Adult Psychiatry (14.4%). The majority of participants identified as being from White British/White other ethnic backgrounds (82.0%), with 7.2% identifying as being from Asian backgrounds and 3.6% from Black African/Caribbean or Black British backgrounds. There were slightly more male participants than female (55.4 vs. 43.4%).

### Views on Existing Treatments for Non-psychotic Mental Illness

Participants overwhelmingly indicated that they felt existing psychotherapeutic and pharmacological treatments for anxiety and depression were either extremely or moderately effective. There was less certainty that existing treatments were effective for substance use disorders, with 49.4% of participants viewing psychotherapeutic treatments for these disorders as only slightly effective or ineffective; and 77.1% viewing pharmacological treatments as slightly effective or ineffective.

### Familiarity With Psychedelics and PAP

All Psychiatrists had heard of at least one psychedelic substance. The most recognised psychedelics were LSD, magic mushrooms, psilocybin (the active component of magic mushrooms) and mescaline. See Table 1 for details.

**Table 1:** Psychiatrists' familiarity with psychedelics substances.

**Name of psychedelic**	**No. of psychiatrists that had heard of psychedelic**	
	**Total *n* = 83**	**%**
LSD	83	100.0
Magic Mushrooms	79	95.2
Psilocybin	78	93.9
Mescaline	71	85.6
Ayahuasca	53	63.9
DMT	49	59.0
Peyote	41	49.4
2c-B	22	26.5
Ibogaine	18	21.7
5-Meo-DMT	11	13.3
DPT	9	10.8
Others	2	2.4
I have not heard of any of these	0	0.0

*Table showing the frequency with which Psychiatrists had heard of various psychedelic substances*.

Around a third of participants (33.7%) believed that psychedelic substances should be decriminalised in the UK, with a fifth (20.5%) believing that the benefits outweighed the harms. Most participants (77.2%) believed that there was a role for controlled or therapeutic use of psychedelics in society.

**Figure 1:**
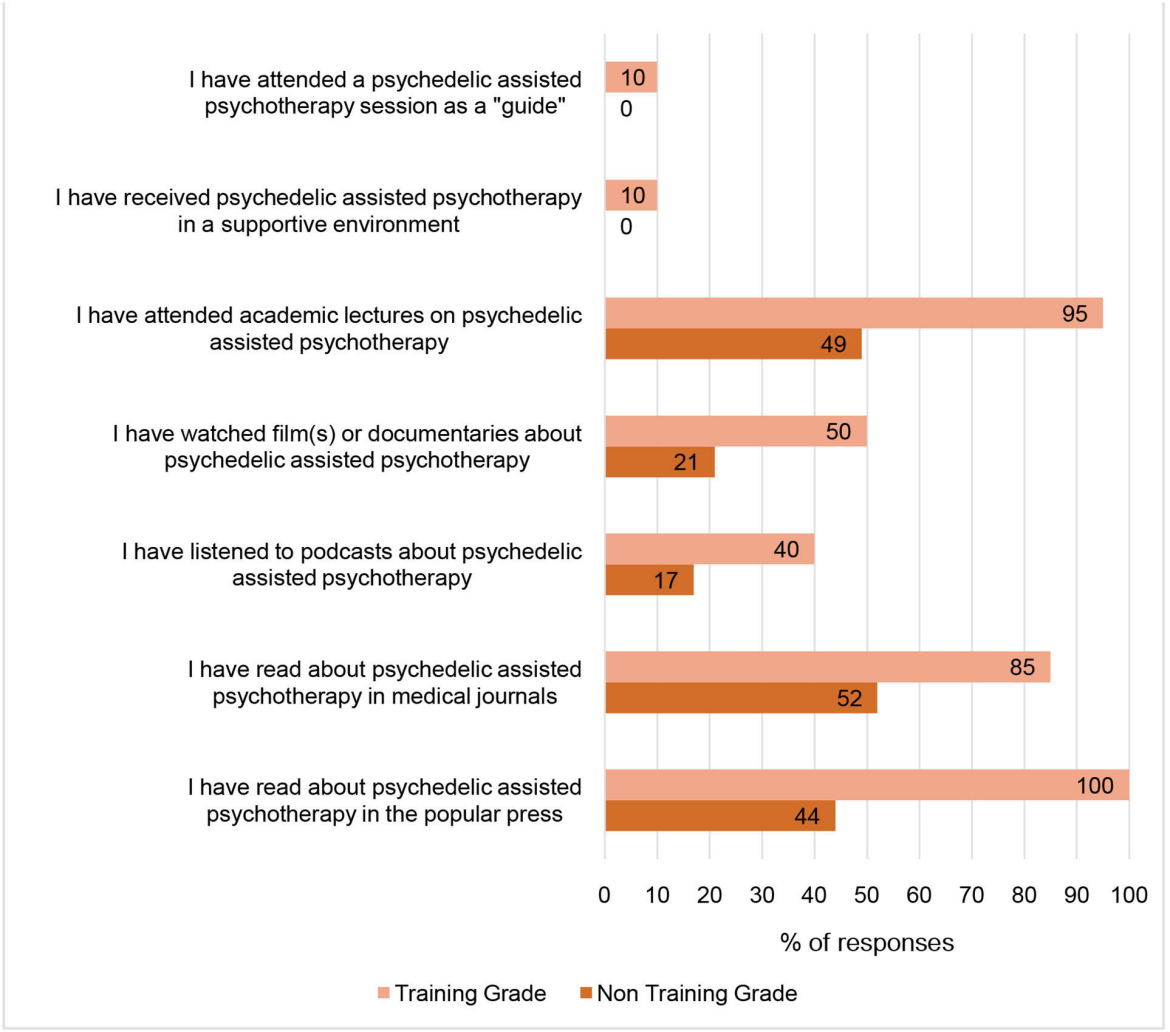
Exposure of psychiatrists to Psychedelic-Assisted Psychotherapy. This figure shows the proportion (%) of psychiatrists who have been exposed to PAP and through which mediums. Total number of responses = 83; Training grade psychiatrists = 20; Non-training grade psychiatrists = 63.

Regarding the therapeutic use of psychedelics, 60.2% were not familiar or only slightly familiar with the use of psychedelics for this purpose, and only 9.6% felt very familiar with this type of use. Only two respondents, who were both trainees, had direct experience of participating in PAP. Overall, trainees demonstrated better knowledge of PAP than non-training grade doctors, with a greater proportion having read journal articles or attended academic lectures. The popular press was also endorsed as a medium by which trainees, in particular, had gained information about PAP. See Figure 1 for how participants gained knowledge about PAP and how this differed between trainees and non-training psychiatrists.

### Involvement With PAP

**Figure 2:**
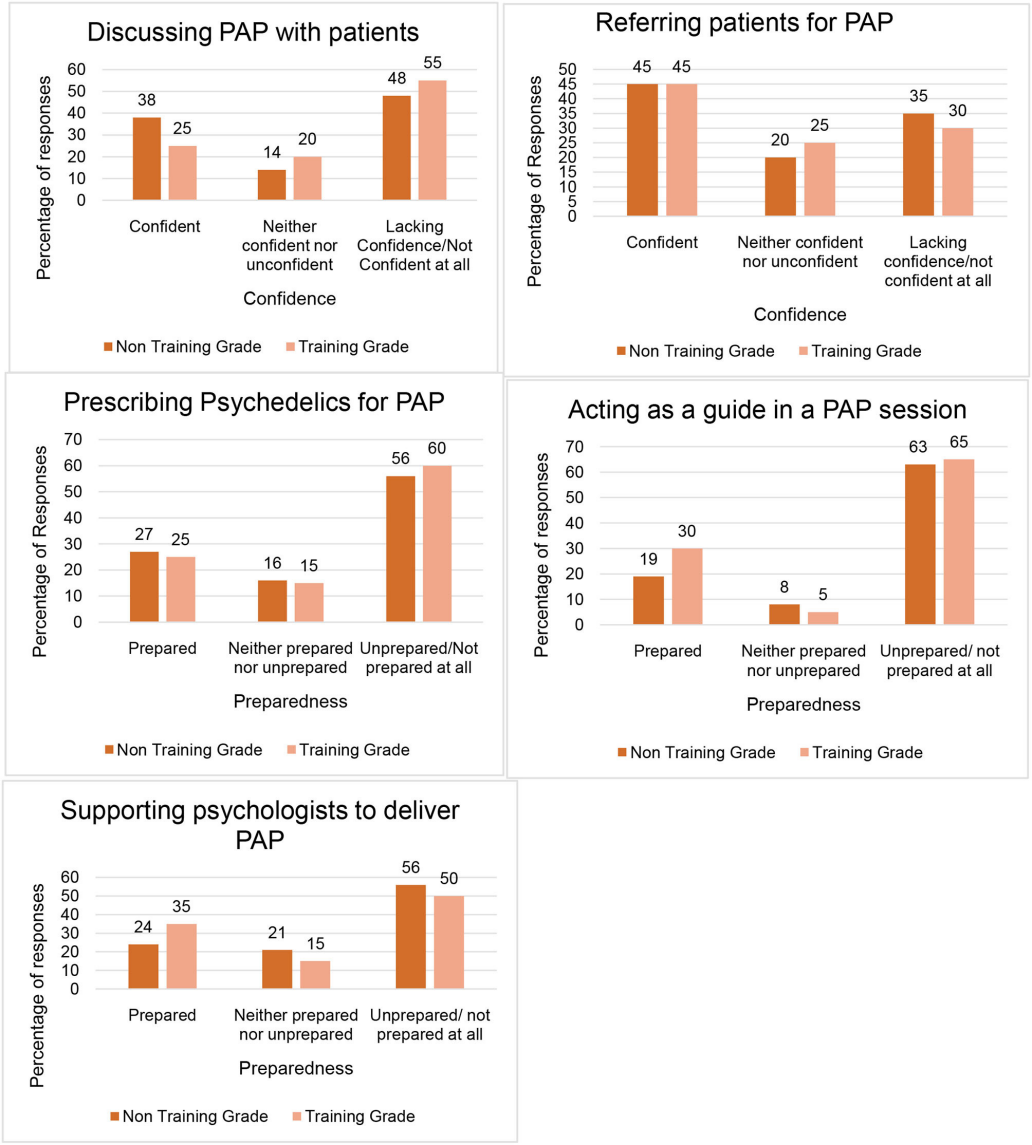
Psychiatrists' confidence and preparedness to be involved with Psychedelic- Assisted Psychotherapy. This figure outlines how confident and prepared Psychiatrists felt to participate in various aspects of PAP. Total number of responses = 83; Training grade psychiatrists = 20; Non-training grade psychiatrists = 63.

Overall, psychiatrists felt unprepared to participate in the practise of PAP; specifically, most did not feel prepared to prescribe psychedelics, act as a PAP guide or support psychologists in the delivery of PAP. Participants indicated a little more confidence to discuss PAP with patients or refer them for PAP treatment. There was little difference between trainees and non-training grade psychiatrists in terms of preparedness. See Figure 2 for how confident and prepared psychiatrists felt to get involved with PAP.

### Psychiatrists' Age Did Not Predict Preparedness or Confidence to Deliver PAP

We had an a-priori hypothesis that younger Psychiatrists would express more confidence in getting involved in the practise of PAP. However, this was not borne out as there was no difference (*p* = 0.78) in the proportion of older (i.e., ≥ 55 years) vs. middle-aged (i.e., 35–54 years) vs. younger (i.e., ≤ 34 years) psychiatrists who felt confident to discuss or refer patients for PAP; to prescribe psychedelics or to support psychology colleagues in the delivery of PAP.

### Focus Groups

The focus groups consisted of 3 or 4 participants in each group—more were expected but there were drop outs on the day. There was a mixture of male and female participants. Participants were from a range of sub-specialties and had differing levels of experience.

**Figure 3:**
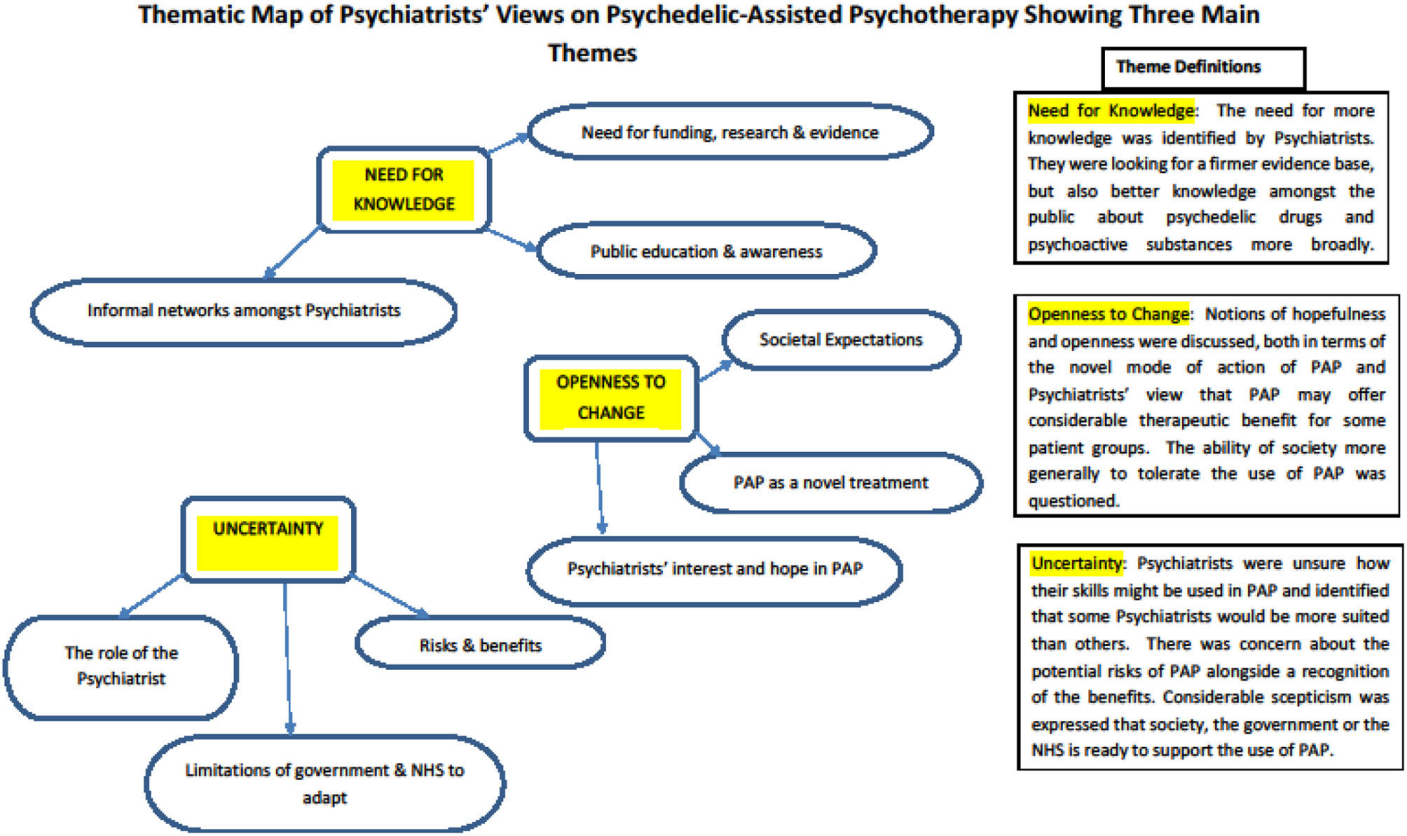
Thematic map detailing the main themes and codes from the thematic analysis of focus groups held with Psychiatrists.

Thematic analysis of the focus group transcripts led to the development of codes and sub-codes. These were iteratively organised and re-organised, from which three overarching themes eventually emerged and were felt to adequately represent the issues that had arisen during the focus groups. Each theme was reviewed and revised until an agreed theme definition was arrived at. The first theme addressed the “*Need for knowledge*” that psychiatrists identified when thinking about the use of PAP; the second theme recognised “*Openness to change*” both within the profession and wider society; and the third theme was one of “*Uncertainty*” for themselves as practitioners, their patients and the frameworks in which they practised. The themes are described in more detail below. A thematic map was developed, which illustrated how each theme was related to its underlying codes—see Figure 3. A table detailing the themes, codes, sub-codes and exemplar extracts is provided in the Supplementary Material accompanying this paper.

### Theme 1: Need for Knowledge

Psychiatrists were seeking a firmer evidence base for PAP and identified a need for well-conducted, funded research on the topic. Much of their own knowledge about PAP had come from their informal networks, rather than from formal sources. Psychiatrists also felt there was a need for better knowledge amongst the general public about psychedelic substances specifically, and psychoactive drugs more generally.

### Theme 2: Openness to Change

Considerable hope and openness towards PAP was expressed. Psychiatrists felt optimistic that PAP had the potential to offer therapeutic benefit to patients for whom little else had worked. They identified PAP as a novel treatment, which could work quickly in the right circumstances. Psychiatrists also wondered about the openness of society to accept or tolerate the therapeutic use of psychedelics.

### Theme 3: Uncertainty

Much uncertainty was expressed. Psychiatrists were unsure how their specific skills might be used in the provision of PAP and there was a range of opinion about how involved psychiatrists should be. There was concern about the potential risks of PAP for some patients, whilst simultaneously recognising the benefits for others. There was scepticism that the structures that support psychiatric practise (i.e., legislative, NHS, professional governance) were ready to support psychiatrists in the delivery of PAP.

## Discussion

Psychedelic-assisted Psychotherapy (PAP) is not yet a treatment that is available beyond the confines of research trials, but interest in its use is growing amongst psychiatric researchers (2, 3) and the public. Assuming the evidence for PAP continues to grow, there will be increasing pressure for PAP to be offered within NHS services. This mixed-methods study was a first attempt to explore knowledge and preparedness amongst practising NHS Psychiatrists about the potential for them to deliver PAP. By using both quantitative and qualitative approaches we were able gain a richness of understanding that would not have been possible had we used only one of these approaches.

The large cross-sectional survey of NHS Psychiatrists of all grades from one large provider mental health trust demonstrated that most psychiatrists are familiar with psychedelic substances and, to some extent, PAP. However, psychiatrists in training appeared better informed about PAP than non-training grade colleagues. Trainees had used a wide range of media to gain knowledge on the topic; this, perhaps, reflects their need to inform themselves generally about advances in psychiatry in preparation for exams—albeit that PAP does not yet feature in their post-graduate curriculum in the UK. The finding that trainees were more familiar with the emerging research was consistent with findings from a previous US study (16), although we did not find an effect of age as they did. Our findings suggested that stigma towards psychedelics was perhaps lower amongst UK psychiatrists, as 77.2% of our study participants felt there was a role for controlled or therapeutic use of psychedelics, whereas around half of the US participants felt that psychedelics were unsafe even in this context.

The survey showed that a substantial minority of NHS psychiatrists felt confident to discuss PAP with their patients or refer them to others for treatment. However, there was a lack of confidence and preparedness amongst psychiatrists to actually deliver or participate in PAP themselves. This is perhaps not surprising given the relatively few modern era clinical trials that have emerged and the legal restrictions that work with psychedelics involves. Our study has highlighted significant training needs for psychiatrists if PAP were to be implemented in NHS practise. Our prediction that younger psychiatrists would feel more confident in the use of PAP was not borne out and training needs are likely to be common across psychiatrists of all seniority and experience.

The thematic analysis of the focus groups enabled a more nuanced exploration of the factors that might be important in providing PAP within NHS settings. The uncertainty surrounding PAP was an important theme and showed that Psychiatrists are not just uncertain about their own role in PAP, but also the uncertain risks and benefits for patients. Recent research from Ireland suggested that just over a half of mental health service users would accept treatment with PAP if a doctor recommended it (18). It seems likely that psychiatrists will be increasingly asked about PAP by patients and be expected to have a working knowledge or who it benefits and how to access it. There was additional uncertainty about how supporting structures around psychiatrists would respond to the introduction of PAP.

The need for more knowledge at multiple levels was emphasised and the need for the public to be educated about the topic prior to its widespread adoption. However, beyond these concerns about how PAP would be conducted and for whom it would be suitable, there was a strong theme of openness to change and excitement at PAP's potential to benefit patients for whom existing treatments have failed. The energy and optimism that (some) psychiatrists identified in relation to PAP would be helpful for services to harness should PAP become a treatment option in NHS clinical practise. PAP is not a straight-forward intervention and to deploy it within existing services will require a focus on implementation and the involvement of enthusiastic psychiatrists.

### Study Strengths and Weaknesses

Strengths of the study included the surveying of all psychiatrists across an entire NHS mental health trust and the involvement of all sub-specialties and levels of experience. The response rate (25.7%) was lower than ideal, although not unusual for a survey of this type. It therefore remains possible that there was response bias, such that psychiatrists who were already more knowledgeable about PAP differentially responded to the survey and were more likely to participate in the focus groups. Certainly, proportionally more trainees participated in the survey than non-training grade doctors, which may have been due to a greater enthusiasm amongst trainees for this topic or may have reflected the greater time pressures on non-training grade doctors. Furthermore, although the sample was likely to be relatively representative of UK psychiatrists—particularly those practising in England—it is not clear that this would hold true for psychiatrists practising in other countries. The mixed-methods approach was an advantage, which is likely to have facilitated richer insights by utilising quantitative and qualitative methods. Nevertheless, more focus groups with more attendees may have elicited additional or different themes, as we could not be certain that saturation of themes was achieved.

The findings of this pilot study suggest that for psychiatrists to develop the necessary expertise and confidence in PAP, they will need support to train and accredit in this new approach. In addition, our findings suggest that getting the support and training structures right for psychiatrists will likely be insufficient unless there is public education about PAP and some degree of societal shift. Formal guidelines, curricula and training—devised in conjunction with regulators and organisations that educate and support psychiatrists—will be needed before psychiatrists will feel ready to participate in the delivery of this promising new treatment paradigm. For example, a multi-disciplinary group from Canada recently recommended that a National Advisory Council be created to make recommendations and around training and accreditation for clinicians involved in the provision of PAP there (19). In summary, however strong the research evidence for the efficacy of PAP in psychiatric disorders, our findings suggest this will be insufficient for PAP to be implemented within NHS mental health services without considerable attention on the workforce needed to deliver it.

## Data Availability Statement

The original contributions presented in the study are included in the article/Supplementary Material, further inquiries can be directed to the corresponding author.

## Ethics Statement

Ethical review and approval was not required for the study on human participants in accordance with the local legislation and institutional requirements. The patients/participants provided their written informed consent to participate in this study.

## Author Contributions

LP conceived and designed the study. AR co-designed, conducted, and analysed the cross-sectional survey. HS and KF convened and facilitated the focus groups. KF and LP analysed the focus groups. HS, AR, and LP contributed to the write up, with LP taking the lead. GC offered oversight, input and supervision into the design of the survey, and focus groups. All authors contributed to the article and approved the submitted version.

## Funding

This work was supported by Brighton and Sussex Medical School and sponsored by Sussex Partnership NHS Foundation Trust. Funding for the publication fees will come from Brighton and Sussex Medical School.

## Conflict of Interest

GC is employed by Small Pharma Inc. The remaining authors declare that the research was conducted in the absence of any commercial or financial relationships that could be construed as a potential conflict of interest. The authors declare that this study received no funding from Small Pharma Inc or any other external organisation.

## Publisher's Note

All claims expressed in this article are solely those of the authors and do not necessarily represent those of their affiliated organizations, or those of the publisher, the editors and the reviewers. Any product that may be evaluated in this article, or claim that may be made by its manufacturer, is not guaranteed or endorsed by the publisher.
