# Supplementary material for: The Readiness of Psychiatrists to Implement Psychedelic-Assisted Psychotherapy
Source: Front Psychiatry. 2021 Nov 26;12:743599. doi: 10.3389/fpsyt.2021.743599 (PMC9231579; doi:10.3389/fpsyt.2021.743599)
Supplement: Supplementary file 1 [file Table_1.docx]

**Table Detailing Themes, Codes, Sub-codes and Exemplar Extracts from the Thematic Analysis of Focus Groups**

| Theme | Code | Sub-codes | Extract |
| --- | --- | --- | --- |
| Need for Knowledge | Need for funding, research & evidence | More funded research  Lacking of knowledge of PAP | “We need more research and people need to know more about the research that’s done.”  “To have money to be able to do a proper study really.”  “It’s difficult to get that evidence because of the difficulty of getting ethics and the use of controlled substances researched.”  “I don’t know almost anything about it.” |
|  | Public education & awareness | People’s minds are closed  The need to address misinformation | “I think it’s starting, but even getting that knowledge out about what’s happening already can be difficult if people’s minds are closed to it.”  “There’s something probably about relatively, looking at the root implications of recreational substances, they are relatively harmless.”  “To kind of demystify these things, there are all these kinds of horrible myths and horror stories from the 70s and people freaking out and never coming back from the dark dimension, the underworld and so forth.”  “Using practical examples and simple language, try to educate the public and say all of the drugs we are dealing with in psychiatry and in the whole of medicine are toxic.”  “Try to shift this public attitude from downright black and white, ‘these are poisons.’ This is a drug like any other drug.” |
|  | Informal networks amongst Psychiatrists | Heard about PAP from friends’ or colleagues | “I have a colleague who does some research in it.”  “I understand there’s some work at Imperial College in this.” |
| Openness to Change | PAP as a novel treatment | Perceived importance for considering novel treatments for treatment-resistant conditions  PAP works differently to current treatments  PAP works faster than standard psychotherapy | “It’s always important to look for new treatment pathways for people with treatment resistance.”  “I have a readiness to think about new treatments for resistance.”  “I think it’s something quite different to what we have.”  “This kind of thing could be disassociated, sort of separated from each other to facilitate rapport and sort of maybe…facilitation of the emotive processes and certain other parts.”  “You can produce results, which you might get from months to years of traditional psychotherapy, in a much smaller number of sessions.”  “If you have psychedelics involved and you are going through a guided process with somebody, people gain insights much quicker.” |
|  | Psychiatrists’ interest and hope in PAP | Interest regarding the potential of PAP  Openness to being involved with PAP | “I’m very curious to find out more about the subject.”  “I hope that over time the research and science overcomes the prejudices.”  “It’s an area that’s being developed so I don’t feel quite as hopeless about it.”  “It feels a little bit hopeless and like it’s impossible and would never happen.”  “I think the road is open for people who would like to do special interests here and there.”  “Speaking to my consultant that I work with about the research, his mind was closed to it. I could just tell it was just a ‘no’ immediately.” |
|  | Societal Expectations | Society’s level of understanding  Societal maturation | “I can imagine sort of the Tom Cruise and the Scientologists getting hold of it.”  “I think there’s a view about mind-bending drugs generally.”  “I do think it has sense in terms of being in a society where we have difficulty accessing and exploring painful memories.”  “I imagine they work as a catalyser to help form connections, being in a society which has very clear ethical or moral values and rules and fear and stigma.”  “Sometimes I wonder if society is ready to hold the responsibility in terms of using drugs to explore in a very mature way. I’m not sure that a big percentage of the society is at that point.”  “I think as long as the economic analyses prove their point, that will overcome institutional resistance.” |
| Uncertainty | Risks & benefits | Adverse effects in some patients  Risks to the Psychiatrist  Clinical benefits | “As the therapy ends the patient might start to wallow in their imaginations.”  “The personality types of patients, which one has to be really wary of; people don’t like certain modalities of psychotherapy for understandable reasons.”  “Those with a history of psychosis and significant dissociative disorders such as complex PTSD, in the conventional sense you try to keep more grounded. One has to be quite careful about this.”  “A friend of mine has a lot of aggression and threats from patients seeking drug treatment and in terms of how you manage that risk, I think that’s a really important thing to think about.”  “Working in forensics there’s obviously a lot of concern about drug use.”  “It disinhibits the person to actually talk about something that wouldn’t otherwise have done.”  “With psychedelics we’re able to put these walls a bit lower and to access more of that ego, that core of what happened to us or the way we perceive our life or our events.”  “There’s these sort of neural pathways that are quite entrenched, and what I heard in that research was about how the experience allows a more open-minded experience and may access to some other ways of seeing it.” |
|  | Limitations of government & NHS to adapt | Reputational problems  Governmental limitations | “Recreational drugs obviously have a very bad name in psychiatry because it causes so many problems.”  “Separating the medicinal use of these substances from the more recreational use of it would be important too…recategorise them as medicinal products rather than party products would be one step I think.” |
|  |  |  | “The government particularly wanted to be seen as hard on drugs.”  “The government is genuinely very reluctant to make any concessions on the use of any kind of dry in any area of psychiatry medicine, they’re just so blinkered.”  “I think it’s more the organisational barrier.” |
|  | The role of the Psychiatrist | Uncertainty around which Psychiatrists should be involved  Leadership role of the Psychiatrist | “What I think is that if you’re going to be a Psychiatrist then you only get a very narrow view…you really have to work in the private sector to widen things out.”  “It depends on what kind of Psychiatrist you are.”    “It might be across specialties.”  “I’m not sure Psychiatrists would necessarily really be the therapist alongside the experience.”  “I’m not sure how active or proactive a Psychiatrist has to be.”  “I think my view is some Psychiatrists are more interested in the psychological aspects of treatment and the psychotherapeutic aspects of treatment…I personally would find that fascinating and really interested to do.”  “I think there’s definitely a role for psychiatry looking at how it’s implemented, how it’s implemented safely, who it should be offered to…I’m thinking more along the lines of governance as well as the sort of everyday clinical decisions.”  “The main role that I could see is assisting with the research and incorporating it into any sort of therapy that the Psychiatrist is doing.”  “I absolutely disagree that they should just be prescribing. It seems to me really important that the Psychiatrist is actually doing the therapy. It may be for practical reasons the Psychiatrists would also be supervising two or three non-medical psychotherapists, and in that case a more prescribing role, but nevertheless following the therapy throughout.”  “Community Psychiatrists or people who have been in long-term contact with patients and know a lot about their history and sort of totality of the presentation, and their history of engagement…from a general point of view.” |
